# Spatiotemporal dynamics of cholera in the Democratic Republic of the Congo before and during the implementation of the Multisectoral Cholera Elimination Plan: a cross-sectional study from 2000 to 2021

**DOI:** 10.1186/s12889-023-16449-2

**Published:** 2023-08-22

**Authors:** Nadège Taty, Didier Bompangue, Nancy Meschinet de Richemond, JJ Muyembe

**Affiliations:** 1 Laboratoire de géographie et d’aménagement de Montpellier, Université Paul Valéry Montpellier 3, Montpellier, France; 2 Service d’Ecologie et Contrôle des Maladies Infectieuses, Faculté de Médecine, Université de Kinshasa, République démocratique, Congo; 3 Programme National d’Elimination du choléra et de lutte contre les autres maladies diarrhéiques, Ministère de la Santé, Hygiène et Prévention, République démocratique, Congo; 4 Laboratory Chrono-Environnement, UMR 6249, University of Bourgogne Franche-Comté, Besançon, France; 5 Institut National des Recherches Biomédicales, Kinshasa, Democratic Republic of the Congo

**Keywords:** Democratic Republic of the Congo, Cholera, Cholera dynamics, Spatiotemporal analysis, Cholera elimination

## Abstract

**Background:**

The Democratic Republic of the Congo (DRC) implemented the first strategic *Multisectoral Cholera Elimination Plan* (MCEP) in 2008–2012. Two subsequent MCEPs have since been implemented covering the periods 2013–2017 and 2018–2021. The current study aimed to assess the spatiotemporal dynamics of cholera over the recent 22-year period to determine the impact of the MCEPs on cholera epidemics, establish lessons learned and provide an evidence-based foundation to improve the implementation of the next MCEP (2023–2027).

**Methods:**

In this cross-sectional study, secondary weekly epidemiological cholera data covering the 2000–2021 period was extracted from the DRC Ministry of Health surveillance databases. The data series was divided into four periods: pre-MCEP 2003–2007 (pre-MCEP), first MCEP (MCEP-1), second MCEP (MCEP-2) and third MCEP (MCEP-3). For each period, we assessed the overall cholera profiles and seasonal patterns. We analyzed the spatial dynamics and identified cholera risk clusters at the province level. We also assessed the evolution of cholera sanctuary zones identified during each period.

**Results:**

During the 2000–2021 period, the DRC recorded 520,024 suspected cases and 12,561 deaths. The endemic provinces remain the most affected with more than 75% of cases, five of the six endemic provinces were identified as risk clusters during each MCEP period (North Kivu, South Kivu, Tanganyika, Haut-Lomami and Haut-Katanga). Several health zones were identified as cholera sanctuary zones during the study period: 14 health zones during MCEP-1, 14 health zones during MCEP-2 and 29 health zones during MCEP-3. Over the course of the study period, seasonal cholera patterns remained constant, with one peak during the dry season and one peak during the rainy season.

**Conclusion:**

Despite the implementation of three MCEPs, the cholera context in the DRC remains largely unchanged since the pre-MCEP period. To better orient cholera elimination activities, the method used to classify priority health zones should be optimized by analyzing epidemiological; water, sanitation and hygiene; socio-economic; environmental and health indicators at the local level. Improvements should also be made regarding the implementation of the MCEP, reporting of funded activities and surveillance of cholera cases. Additional studies should aim to identify specific bottlenecks and gaps in the coordination and strategic efforts of cholera elimination interventions at the local, national and international levels.

**Supplementary Information:**

The online version contains supplementary material available at 10.1186/s12889-023-16449-2.

## Background

Cholera is a highly contagious diarrheal disease caused by toxigenic strains of *Vibrio cholerae* serogroups O1 and O139 [1]. The disease is primarily contracted by ingesting water or food contaminated with the bacterium. Cholera symptoms include diarrhea, sometimes accompanied by vomiting, which occurs within hours to five days after infection [2]. Without treatment, acute dehydration induced by the disease results in death within hours in more than half of cases. Case management, which consists mainly of rehydration, significantly reduces the mortality rate to less than 1% [3].

A total of seven cholera pandemics have been documented, the current and longest of which has been raging for over 60 years [4]. Continental Africa has been affected by the disease since the 1970s [5]. The first cases of cholera in the Democratic Republic of the Congo (DRC) were officially declared in 1973, with cases introduced from Angola to Kongo Central Province in western DRC. In 1977, cases were introduced from Tanzania to Tanganyika Province in eastern DRC [6]. Since 1977, cholera has remained a public health threat in the country, with cholera cases notified to the World Health Organization (WHO) every year. The DRC has reported approximately 5–14% of global cholera cases, with hundreds of deaths that could be prevented each year [7]. In 2017, the DRC recorded the largest cholera epidemic since the year 2000, with more than 53,000 suspected cases and 2,300 deaths [8]. In 2019 and 2020, the DRC was second in the world after Yemen and first in Africa in terms of cholera burden, with 55% of cases and 55% of deaths in 2019 [9] and 41.8% of cases and 47.7% of deaths in 2020 [10].

The Global Task Force on Cholera Control (GTFCC) has established the *Global Roadmap to 2030* strategy, which aims to reduce cholera deaths by 90% and eliminate the disease as a public health threat in affected countries by the year 2030 [11]. To achieve cholera elimination, the multidisciplinary package of activities should include strengthened disease surveillance with early detection and rapid response; coordinated control activities; quality and rapid case management; improved water supply, sanitation and environmental hygiene; social mobilization; behavior change education (collective hand and food hygiene); and oral cholera vaccination as a complementary measure [12].

The DRC is one of the first countries in the world to have developed a strategic plan to eliminate cholera based on the results of eco-epidemiological studies conducted in the country from 2005 to 2007. The first strategic *Multisectoral Cholera Elimination Plan* (MCEP) was implemented in 2008 to better manage this health crisis and stop cholera transmission nationwide, with an annual threshold of one culture-confirmed case per 1,000,000 inhabitants, i.e., less than 500 new confirmed cases per year [13]. To date, three MCEPs have been implemented in the DRC. After the mixed success of the first plan (2008–2012) due to a lack of funding, the second plan (2013–2017) obtained more investment, including targeted development activities such as drinking water supply projects in two cholera foci: Kalemie and Uvira [14]. The third plan covers the period from 2018 to 2021 [15].

Cholera elimination strategies should be guided by an in-depth understanding of local cholera dynamics. Studies conducted in the Great Lakes region of eastern DRC have highlighted the seasonal patterns of cholera epidemics and identified certain health zones where the disease persists during relative lull periods. The studies have also identified specific at-risk and mobile communities in these health zones, such as fishermen and merchants. These health zones in eastern DRC thus play a major role in maintaining cholera outbreaks and diffusing the disease throughout the country [16, 17].

To eliminate cholera in the DRC by the year 2030, activities are focused in the six endemic provinces: Ituri, North Kivu, South Kivu, Tanganyika, Haut Lomami and Haut Katanga. In these provinces, the following health zones have been identified as priority cholera sanctuary zones in 2003–2007: Tshomia (Lake Albert), Goma and Bukavu (Lake Kivu), Uvira-Fizi (extreme north of Lake Tanganyika), Kalemie (center of Lake Tanganyika), Kilwa and Pweto (Lake Moero), and Bukama-Kinkondja-Malemba Nkulu (Lake Upemba-Kaziba) [16]. The MCEP aims to provide sustainable and structural solutions to address the factors responsible for cholera persistence and diffusion in these health zones.

The current study aimed to assess the spatial and temporal dynamics of cholera before the implementation of the elimination plan and during each MCEP period to determine the effect of the elimination plans on cholera dynamics at the provincial and health zone levels, establish limitations and lessons learned, and provide an evidence-based foundation to guide the next MCEP (2023–2027). Overall, these findings will serve to strengthen the efforts to eliminate cholera in the DRC by the year 2030.

## Methods

### Study design and site

We conducted a cross-sectional study of cholera outbreaks in the DRC using weekly national surveillance data of suspected cholera cases and deaths from 2000 to 2021. We also analyzed biological data of clinical *Vibrio cholerae* isolates obtained from the National Institute of Biomedical Research (NIBR) from 2015 to 2021. We reviewed the three MCEPs developed in the DRC and the evaluation reports of the first two MCEPs.

The DRC is located in the heart of Africa and covers an area of 2,345,000 km^2^. The country is divided into 26 provinces (Additional file 1) and 518 health zones. In 2020, the country had an estimated population of 92,853,164 inhabitants [18]. The Congo River crosses almost the entire country from east to west over 4,700 km. The DRC is situated on the equator and thus has the full range of climate characteristics of the humid tropical zone: an equatorial climate in the center, a tropical and humid climate in the north and south, a temperate climate at high altitudes in the east and a mountainous climate in the extreme east from Lake Albert to Lake Kivu. In general, the seasons in the DRC are as follows: January to February, short dry season; March to May, short rainy season; June to September, long dry season; and October to December, long rainy season.

### Surveillance data sources

The weekly epidemiological data from 2000 to 2021 were obtained from the DRC Ministry of Health. The Integrated Disease Surveillance and Response (IDSR) system has been in place in the DRC since 2000, managed by the Ministry of Health in collaboration with WHO. The IDSR system is a syndromic surveillance system that compiles weekly reports of morbidity and mortality, aggregated at the health zone level. The IDSR system covers thirteen diseases with epidemic potential, including cholera, for passive surveillance outside of epidemic periods. Passive surveillance is coupled with active surveillance during epidemics. The IDSR system uses two definitions for suspected cholera cases depending on whether a cholera epidemic has been declared by the Ministry of Health (IDSR, 3rd edition):

- In areas without a declared cholera epidemic: any patient two years of age and older with acute watery diarrhea and severe dehydration or death due to acute watery diarrhea.
- In areas where a cholera epidemic has been declared: any person presenting with or dying of acute watery diarrhea.

### Identification and classification of cholera sanctuary zones

The method used to identify and classify cholera sanctuary zones has been previously described [19]. Briefly, cholera sanctuary zones were identified at the health zone level based on epidemiological parameters (number of suspected cases notified per week, persistence of suspected cholera cases and attack rate per 100,000 inhabitants) and environmental indicators (proximity to the lake; presence of a lake, port or road in the health zone). Cholera sanctuaries notify cholera cases in a quasi-continuous manner, with lull periods (reporting zero cases per week) of < 16 weeks. In these areas, cholera outbreaks can resurge and spread to other nearby areas.

Cholera-endemic provinces report cholera cases in a continuous or metastable manner. These provinces have at least one cholera sanctuary according to the MCEP 2018–2021 classification. During the MCEP-3 period, six provinces were considered cholera endemic (Ituri, North Kivu, South Kivu, Tanganyika, Haut Lomami and Haut Katanga), and in these provinces, 29 health zones have been identified as cholera sanctuary zones [15].

Non-endemic provinces report cholera cases in a recurrent (> 3 outbreaks in the previous 5 years), intermittent (< 3 outbreaks in the previous 5 years) or sporadic (1 outbreak in the previous 5 years) manner.

### Microbiological data

Biological confirmation data were obtained from the national cholera reference laboratory at the NIBR. The NIBR performs routine culture confirmation tests for national surveillance and epidemic confirmation (data available from 2015 to 2021). Fecal samples or rectal swabs from suspected cholera cases, which are typically collected at the beginning, middle, and end of a cholera outbreak, are placed in Carry-Blair transport medium and transported to the NIBR for culture confirmation. For each sample tested at the NIBR, the following data were collected: year, number of samples taken and number of positive results.

### Population data

To calculate cholera incidence and attack rates, we used population estimates at the health zone and province levels from 2000 to 2021, which was obtained from the Expanded Program on Immunization considering a stable population growth of 1.03% [20]. This growth rate has also been consistency applied in similar analyses of other diseases [21–23].

### Data organization and analysis

Secondary weekly epidemiological data were extracted from the DRC Ministry of Health surveillance databases from 2000 to 2021. The extracted data were cleaned and analyzed for weekly cases and deaths using Microsoft Excel and R software (R packages rcmdr, ggplot and MASS).

To analyze the data throughout the study period, we applied the administrative divisions that were adopted in 2015 (26 provinces, instead of the previous 11 provinces) for the 2000–2014 data [24]. Epidemic curves at the national and provincial levels were produced to assess the temporal evolution of cholera epidemics before and during the implementation of each MCEP. Weekly and annual changes in case fatality rates were calculated with cholera deaths as the numerator and cholera cases as the denominator. To compare the periods before and during the implementation of each MCEP, the data series was divided into four periods: the pre-MCEP period 2003–2007 (pre-MCEP), the first MCEP period 2008–2012 (MCEP-1), the second MCEP period 2013–2017 (MCEP-2), and the third MCEP period 2018–2021 (MCEP-3). The averages of these four periods were compared using ANOVA. To perform the ANOVA, we first tested the data for normal distribution using the Chapiro-Wilk test [25], which indicated that the data are normally distributed (threshold: *p* > 0.05) [17]. We then verified variance homogeny using the Bartlett test, indicating that the data are statistically homogeneous (threshold: *p* > 0.05) [26]. We used the confidence interval method according to which the means of the two groups are not different when the confidence intervals overlap.

We identified seasonal patterns by decomposing the weekly time series using R software as described by Cleveland et al. [27]. This analysis was performed on three five-year time series (pre-MCEP, MCEP-1 and MCEP-2) and one four-year time series (MCEP-3).

### Cartography

To assess the spatial dynamics of cholera cases, we produced maps of average attack rates for the four periods (pre-MCEP, MCEP-1, MCEP-2 and MCEP-3) at the province level. Average attack rates were calculated as follows: average sum of cases for the period*100,000 /average population for the period. The calculated attack rates are expressed as cholera cases per 100,000 inhabitants. Attack rate maps were generated using QGIS 3.16 Madeira software with shapefiles obtained from www.DIVA-GISgis.org/gdata.

### Cluster analysis

For each time period, we performed a retrospective cluster analysis using a Poisson-based space-time permutation scan statistic according to Kulldorff et al. [28] with SaTScan software version 9.6. We identified risk clusters at the provincial level with a reactive risk > 1 and a p-value > 0.05 [29]. These risk clusters were then mapped using QGIS 3.16 software.

## Results

### Epidemiological description of cholera cases and deaths

**Fig. 1:**
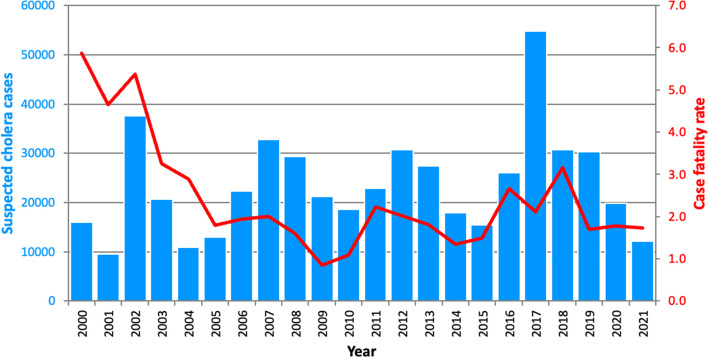
Annual number of suspected cholera cases and case fatality rate in the DRC from 2000 to 2021

From 2000 to 2021, the DRC recorded 520,024 suspected cases and 12,561 deaths, representing a case fatality rate of 2.4% (Fig. 1). Every province in the country and 498 of 518 (96%) health zones reported suspected cholera cases. In 2017, the DRC recorded the highest annual number of cholera cases since 2000, with more than 53,000 suspected cholera cases (Fig. 1). During the study period, the average annual case fatality rate was above 1%, thus potentially indicating suboptimal or delayed medical treatment.

**Fig. 2:**
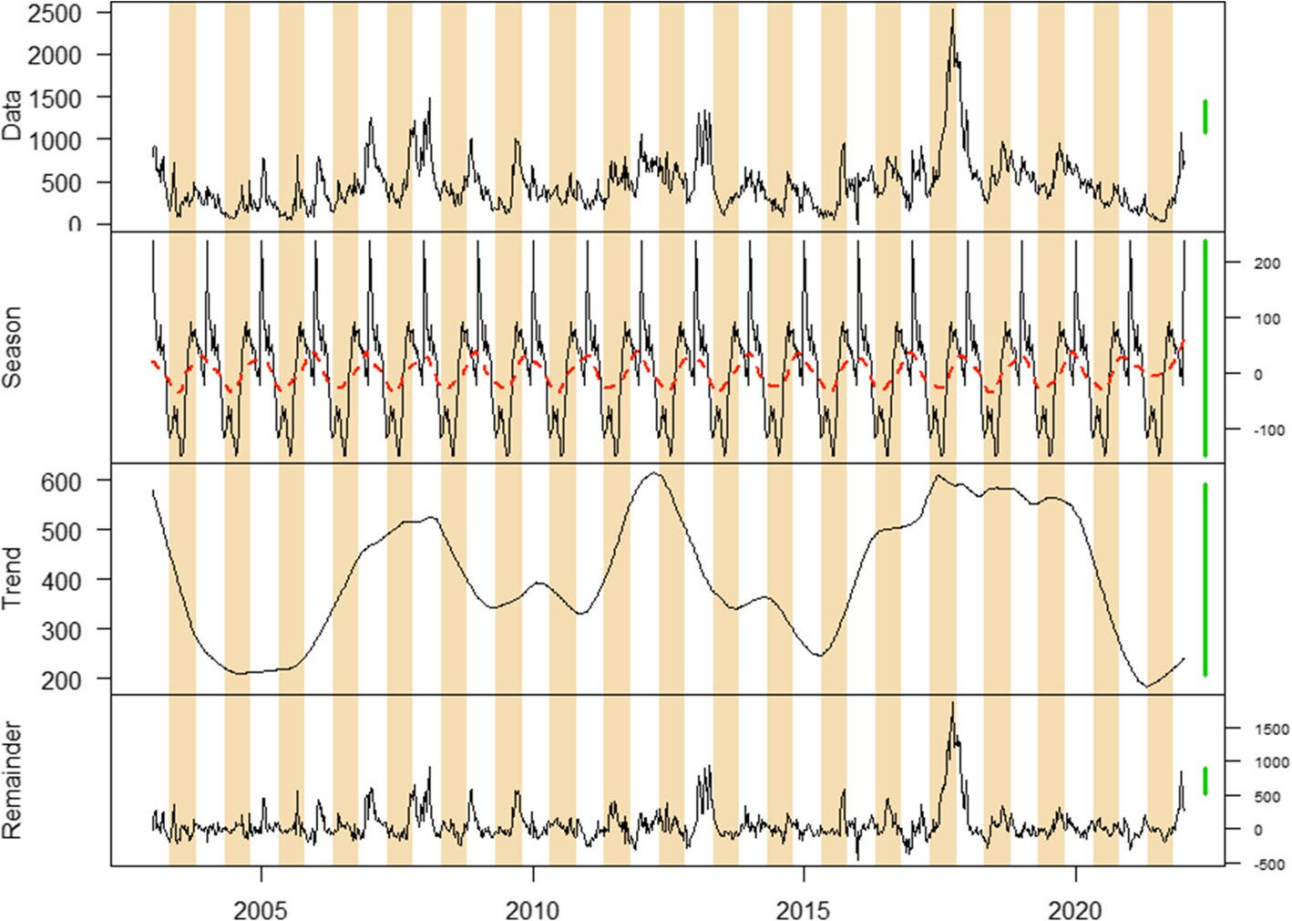
Seasonal cholera outbreak patterns in the DRC from 2003 to 2021. Decomposition of time series into three components: season, trend and remainder. The alternating white and tan stripes represent the rainy and dry seasons, respectively

At the national level, we did not observe a significant difference in terms of numbers of cases and deaths before and during the implementation of each MCEP (p-value > 0.05) (Table 1). During the pre-MCEP, MCEP-1 and MCEP-2 periods, the seasonal cholera patterns remained the same, with two epidemic peaks: a small peak towards the end of the dry season and a large peak during the middle of the rainy season. However, during the MCEP-3 period, a large epidemic peak was observed at the end of the dry season and a small peak occurred during the rainy season (Fig. 2).

**Table 1:** Comparison of average cholera case numbers during each period

Periods compared	Difference in average case numbers	Std. error	t value	Pr(>|t|)
MCEP-1 – Pre-MCEP	968.5	2431.8	0.398	0.978
MCEP-2 - Pre-MCEP	1691.9	2431.8	0.696	0.898
MCEP-3 - Pre-MCEP	-358.5	2431.8	-0.147	0.898
MCEP-2 – MCEP-1	723.4	2431.8	0.297	0.991
MCEP-3 – MCEP-1	-1327.0	2431.8	-0.546	0.947
MCEP-3 – MCEP-2	-2050.3	2431.8	-0.843	0.834

Adjusted *p*-values reported - single-step method

### Biological results

From 2015 to 2021, a total of 7,518 stool samples were collected from 189,165 suspected cholera cases (collection rate: 3.9%). The highest collection rate was in 2011 (11.6%), while the lowest collection rate was in 2018 (1.3%). The culture positivity rate was 25.9% (positivity rates ranged from 43.5 to 11.1%) (Table 2).

**Table 2:** Distribution of suspected cholera cases, number of stool samples taken and number of positive culture samples

Year	Total suspected cholera cases	Number of stool samples sent to the NIBR	Number of stool culture samples that tested positive for *Vibrio cholerae* O1
2015	15,444	1,809 (11.7%)	696 (38.4%)
2016	25,982	1,238 (4.7%)	252 (20.3%)
2017	54,779	1,000 (1.8%)	217 (21.7%)
2018	30,768	411 (1.3%)	146 (35.5)
2019	30,304	514 (1.6%)	224 (43.5%)
2020	19,785	1,340 (6.7%)	283 (21.1%)
2021	12,103	1,205 (9.9%)	134 (11.1%)
**Total**	**189,165**	**7,518 (3.9%)**	**1,952 (25.9%)**

### Epidemiological description of cholera patterns in the endemic and non-endemic provinces in the DRC before and during the implementation of the MCEPs

**Fig. 3:**
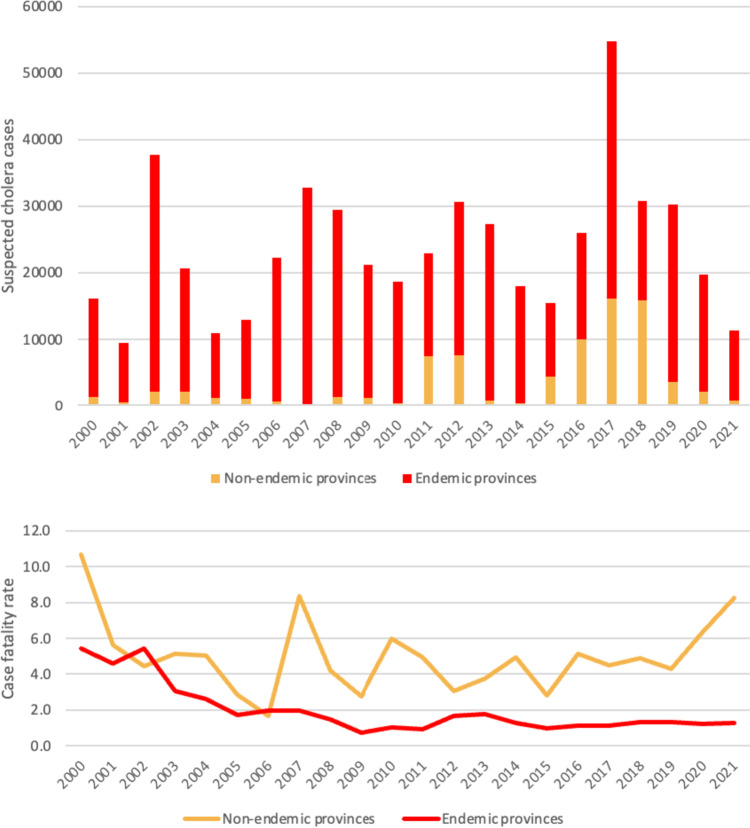
Trends in annual suspected cholera case numbers (upper panel) and case fatality rates (lower panel) in cholera endemic and non-endemic provinces in the DRC

The highest proportions of cases were recorded by the endemic provinces, while the highest case fatality rates were reported by the non-endemic provinces. The endemic provinces recorded 438,888 suspected cases (84.4%) with a case fatality rate of 2%. The mortality rate in endemic provinces has gradually decreased, which may be due to better capacity and preparedness in terms of case management and/or improved awareness and healthcare seeking behavior among at-risk populations. Meanwhile, the non-endemic provinces reported 81,135 suspected cases (15.6%) with a case fatality rate of 4.5% (Fig. 3).

**Fig. 4:**
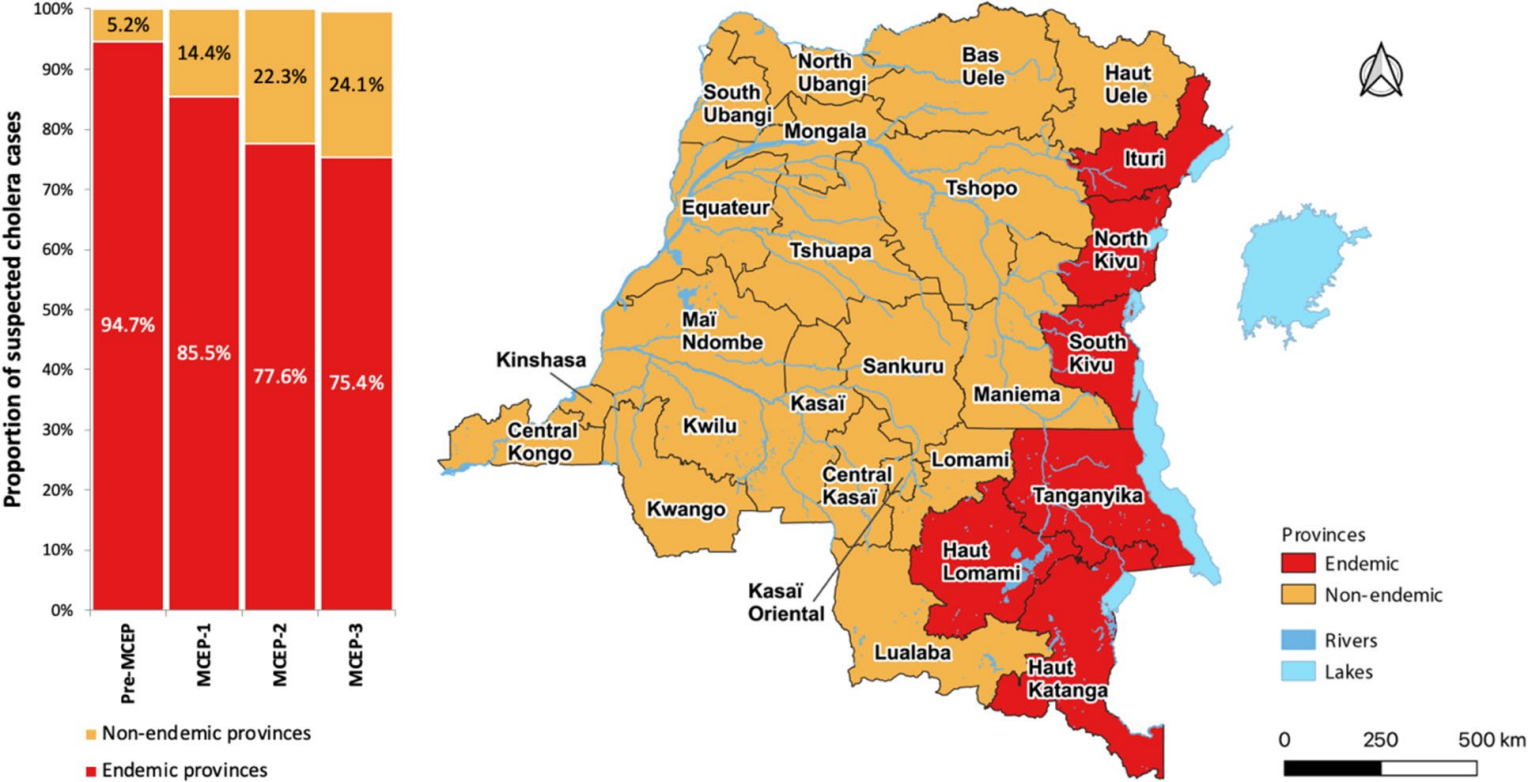
Evolution of the proportion of suspected cholera cases in endemic and non-endemic provinces before plan implementation and during each MCEP period

At least 75% of cases during each period (pre-MCEP, MCEP-1, MCEP-2 and MCEP-3) were recorded by the endemic provinces, i.e., 94.7%, 85.5%, 77.6% and 75.8%, respectively. However, over the course of the study period, we found that the proportion of cases in endemic provinces decreased over time, while the proportion of cases in non-endemic provinces increased (Additional file 2 and Fig. 4).

From 2000 to 2021, more than half of the suspected cholera cases in the endemic provinces were recorded by the provinces of South Kivu and North Kivu, with 30.1% and 25.3% of cases, respectively, followed by Tanganyika (16.1%), Haut-Lomami (13.9%), Haut Katanga (10.5%) and Ituri (3.8%). Over the course of the study period, the provinces of Tanganyika and South Kivu had the highest attack rates. Tanganyika had attack rates > 100 cholera cases per 100,000 inhabitants during all four periods. South Kivu had attack rates > 100 during the pre-MCEP and MCEP-1 periods and attack rates of 50–100 during the MCEP-2 and MCEP-3 periods (Additional file 3).

**Fig. 5:**
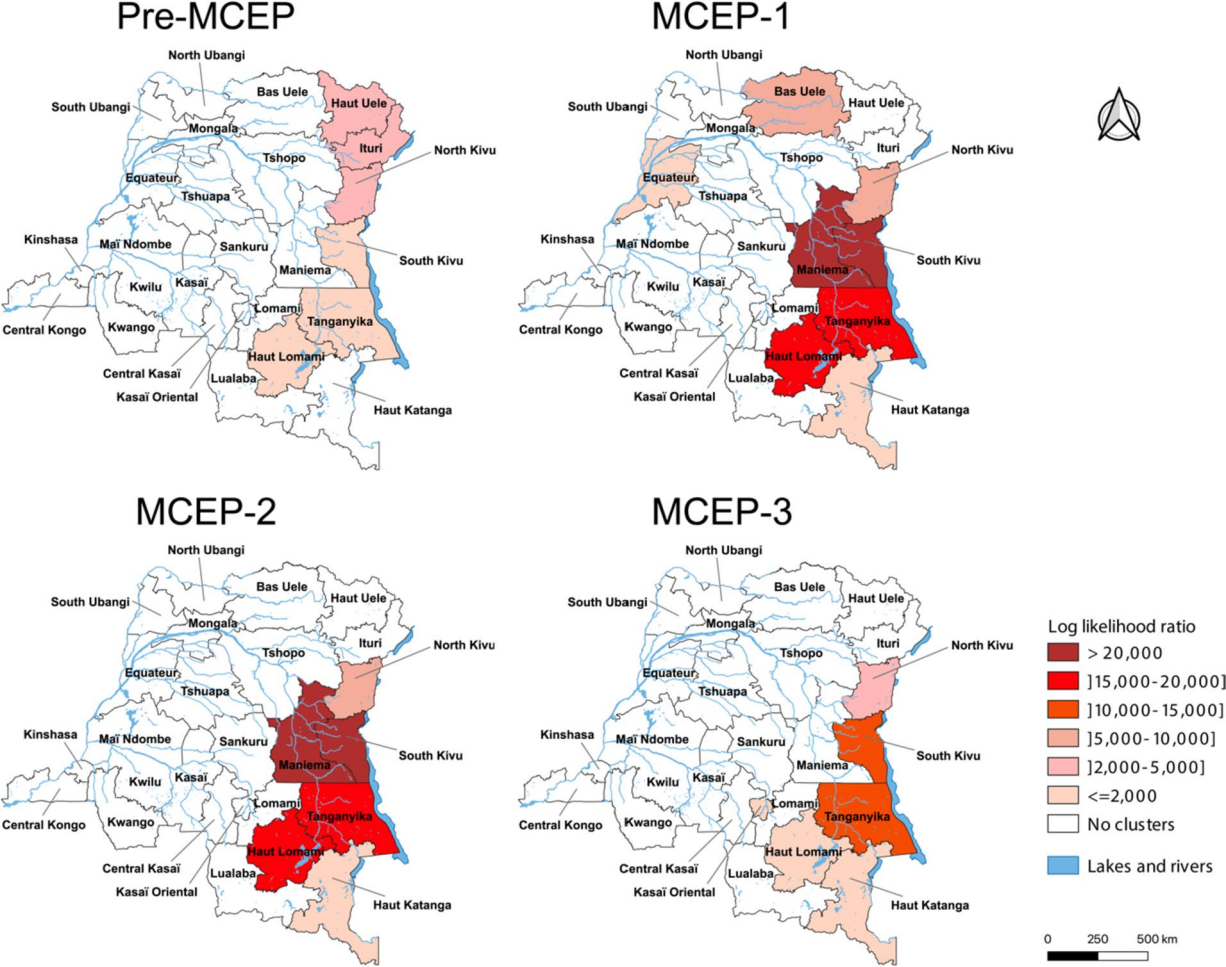
Cholera risk clusters in the DRC at the provincial level, before and during each MCEP period

Province-level cholera risk clusters were largely concentrated in endemic provinces. During the pre-MCEP period, clusters were identified in five endemic provinces and one non-endemic province (Haut-Uélé); the risk was highest in the northeastern provinces. After the implementation of the MCEP, Ituri Province was the only endemic province not identified as a risk cluster. Apart from the endemic provinces, four non-endemic provinces were identified as risk clusters: Maniema (MCEP-1 and MCEP-2), Bas-Uélé (MCEP-1), Equateur (MCEP-1) and Kasaï-Oriental (MCEP-3) (Fig. 5).

### Evolution of cholera epidemiology in the endemic provinces

**Fig. 6:**
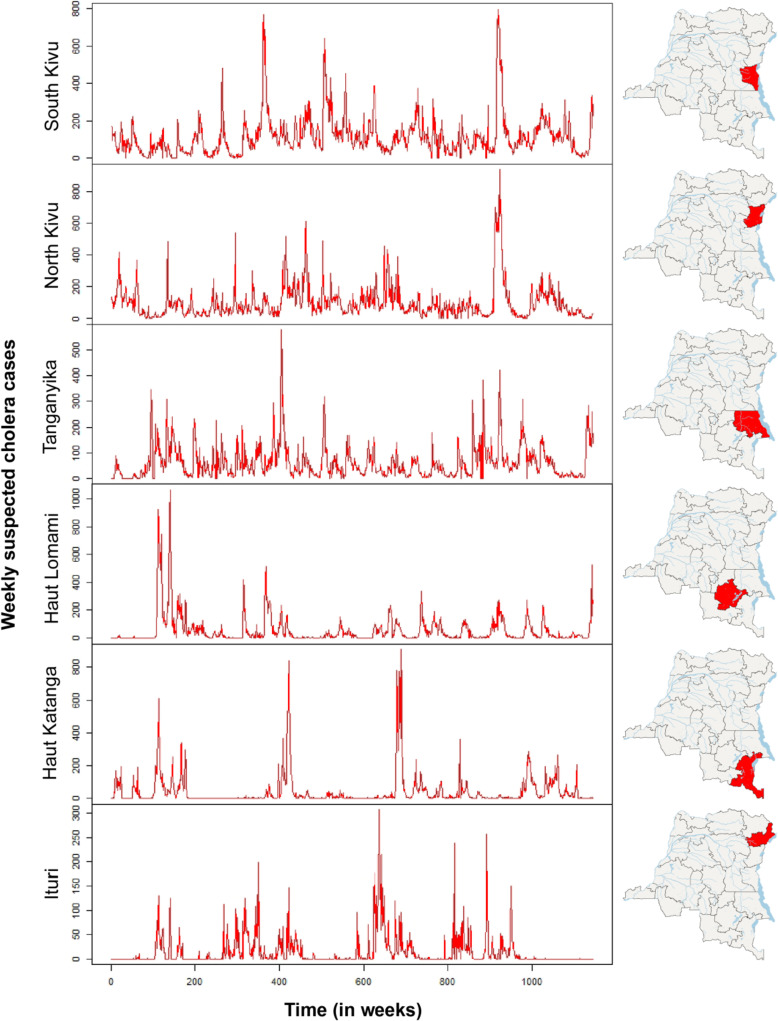
Weekly number of cholera cases in cholera-endemic provinces, 2000–2021

Over the entire study period, three endemic provinces (North Kivu, South Kivu and Tanganyika) recorded cases almost continuously. Tanganyika never recorded an interruption in cases of more than four weeks, while North Kivu and South Kivu each recorded a single five-week interruption during the MCEP-2 period (Fig. 6 and Additional file 4).

### Evolution of health zone cholera profiles during the three MCEP periods

A total of 14 health zones were identified as cholera sanctuary zones during MCEP-1, 14 sanctuary zones were identified during MCEP-2, and 29 sanctuary zones were identified during MCEP-3 (Table 3). Six health zones were considered cholera sanctuary zones during all three MCEP implementation periods: Kalemie and Nyemba (Tanganyika), Goma and Karisimbi (North Kivu) and Uvira and Kadutu (South Kivu). These health zones recorded 24.2% of all suspected cholera cases during the study period. Four health zones identified as sanctuary zones during the MCEP-1 period were no longer considered sanctuary zones during the two subsequent MCEPs: Ibanda and Bagira (South Kivu), Kasenga and Pweto (Haut-Katanga). Sanctuary zones identified during the MCEP-3 period included all 14 sanctuary zones during the MCEP-2 period (representing 45% of the cases reported during the MCEP-3 period), 11 new health zones and four health zones identified during MCEP-1.

**Table 3:** Health zones identified as cholera sanctuaries during each of the three MCEP periods

Province	Health zone	MCEP-1	MCEP-2	MCEP-3
Haut Katanga	**Kasenga**	X		
	**Kilwa**	X		X
	**Pweto**	X		
Haut Lomami	**Bukama**	X		X
	**Butumba**	X		X
	**Kabondo-Dianda**			X
	**Kinkondja**		X	X
	**Malemba-Nkulu**		X	X
	**Mukanga**			X
Ituri	**Angumu**			X
	**Bunia**	X		X
	**Mahagi**			X
	**Nyarambe**			X
	**Tchomia**		X	X
North Kivu	**Goma**	X	X	X
	**Karisimbi**	X	X	X
	**Kirotse**		X	X
	**Masisi**			X
	**Mutwanga**			X
	**Mweso**		X	X
	**Nyiragongo**			X
	**Rutshuru**			X
South Kivu	**Bagira**	X		
	**Fizi**		X	X
	**Ibanda**	X		
	**Kadutu**	X	X	X
	**Katana**			X
	**Minova**		X	X
	**Nundu**			X
	**Uvira**	X	X	X
Tanganyika	**Kalemie**	X	X	X
	**Moba**		X	X
	**Nyemba**	X	X	X
**Total per period**		14	14	29

## Discussion

The retrospective analysis of cholera surveillance data from 2000 to 2021 revealed that this disease remains a public health threat in the DRC since 2008 despite the implementation of the MCEP. During the study period, the DRC recorded 520,024 cases and 12,561 deaths (case fatality rate of 2.4%). Over the 22-year period, all provinces and 498 of 518 (96%) health zones reported suspected cholera cases.

Although the endemic provinces recorded more than 75% of all cases during each period, the proportion of cases in the endemic provinces decreased over time, while the proportion of cases in the non-endemic provinces increased. The high case fatality rate of 2.4% [30] may be due to delays in the surveillance system to trigger a rapid response [31] and/or delayed access to healthcare facilities among patients because of insufficient knowledge about cholera or distance from health centers [32]. This case fatality rate varied according to the level of endemicity, from 2% in endemic provinces to 4.5% in non-endemic provinces. The high case fatality rates observed in the non-endemic provinces could be due to low levels of immunity, suboptimal treatment, and the absence of preparedness and prevention activities [33].

At the national level, we did not observe a significant difference in terms of the number of cases and deaths before and during each MCEP period (p-value > 0.05). This lack of progress in cholera elimination may be due to limitations in the method used to identify and prioritize sanctuary zones, which focused essentially on epidemiological indicators (persistence of suspected cholera cases and attack rate per 100,000 inhabitants) and some environmental indicators (proximity to the lake; presence of a lake, port or road in the health zone, etc.). The classification of the health zones in the DRC did not take into account social factors, population displacement, conflict and other factors that may impact disease transmission. Indeed, conflict can further drive cholera diffusion and hinder outbreak response efforts [34], and a recent study has found that conflict increased cholera risk in the DRC by 2.6 times [35]. Populations forcibly displaced by conflict (or other crises) are also often affected by infectious diseases such as cholera [36]. Furthermore, the Global Task Force on Cholera Control recommended that countries integrate water, sanitation and hygiene (WASH) indicators and contextual factors to identify at-risk areas, but only in locations where cholera transmission is low [37].

To better orient epidemic prevention and preparedness activities, health zones should be classified and prioritized based on epidemiological, WASH (e.g., access to drinking water and hygienic toilets), socio-economic, environmental and health indicators (e.g., number of doctors, number of nurses, rate of attendance at health facilities and rate of chronic malnutrition) [38, 39]. The health zone classification should also be updated as needed to assess evolving cholera dynamics in the country and monitor the cholera elimination progress and lessons learned in each health zone. Furthermore, subsequent studies should also investigate the factors that influenced the reduction in case numbers in Ituri Province as well as the increase in health zones classified as sanctuary areas over the course of the study period.

The lack of significant progress in cholera elimination in the DRC may also be due to poor management of the epidemic response, including limits in the development of the MCEP, failure to adhere to established strategies and substandard implementation in the field by the various actors. Indeed, the cholera context in the DRC has not improved in recent years despite significant advances in the understanding of environmental cholera dynamics [40], the establishment of effective community-based control strategies [41] and new tools to control cholera such as vaccination [42]. In the current study, we observed a clear seasonal pattern with two epidemic peaks: one peak towards the end of the dry season and one peak in the middle of the rainy season. Previous cholera studies conducted in the DRC from 2000 to 2007 have identified the same seasonal pattern [43]. This seasonal characteristic should enable actors to anticipate cholera outbreaks and plan preparedness and response activities accordingly. However, during the large-scale epidemic in 2017 (which started during the dry season), field investigations revealed that limited prevention and preparedness activities had been carried out. The epidemic in 2017 started in a few areas where outbreaks persisted (Goma City in the east, Kimpese Health zone in the southwest and the health zones of Bolobo and Bandundu in the northwest) and then spread to more than half of all health zones in the country. This epidemic occurred in the context of a 253% increase in annual cholera case numbers throughout the African continent from 2016 to 2017 [44]. The anticipation and rapid containment of this epidemic could have prevented this health crisis and countless avoidable deaths [45]. Control efforts largely involved punctual response activities with little to no preparedness activities. Furthermore, once case numbers subside, the end of the outbreak is often poorly managed. Additional studies on the cholera outbreak response in the DRC should aim to identify bottlenecks and gaps at the local, national and international levels [46].

The DRC still faces major challenges to eliminate cholera, including coordination of MCEP interventions, orienting partners towards priority sites, monitoring and reliance on external assistance. To ensure that the plan is effectively implemented, the Congolese government should be the primary financer of the MCEP, play a leading role in the implementation of the plan, and channel the funds of all partners involved in the response appropriately, while maintaining an overview of all funds received by each actor. Several evaluations of the MCEPs have been carried out, although these reports do not include an economic evaluation. Nevertheless, all evaluation reports have indicated insufficient funding as a major obstacle to implement the MCEP [47]. To achieve cholera elimination in the DRC, all actors involved in the MCEP must be aligned and all implicated ministries (Ministry of Planning, Ministry of Energy, Ministry of the Environment and the Ministry of Fisheries and Livestock) should be actively involved in the implementation of the plan.

Even with limited resources, cholera elimination can be achieved by applying a multidisciplinary and coordinated approach, with targeted prevention and control activities based on solid scientific evidence and adapted to local contexts [41]. In 2012, Haiti established a cholera elimination plan that was inspired by the DRC’s cholera management strategy [48]. Thanks to the highly coordinated efforts, this country did not report a single confirmed case for over three years, from the beginning of February 2019 to October 2022 [49–51]. However, the Haitian Ministry of Public Health and Population reported two confirmed cholera cases and several suspected cases in the Port-au-Prince metropolitan area on October 2, 2022 [50]. Cholera has since resurged in the country in the context of a complex humanitarian crisis with major socio-political unrest [52–55]. Sanctuary zones and large cities at high risk of cholera outbreaks in the DRC should be prioritized for extended water network projects, and cholera vaccination strategies should be optimized. Furthermore, control strategies must be flexible. For example, although the WHO recommends combining antibiotics to treat severe cases [3], phagotherapy may serve as an alternative to antibiotics, as cases of antibiotic resistance have increased in the DRC [56].

To declare the elimination of cholera in the DRC, the Congolese government has set the threshold at one culture-confirmed case per 1,000,000 population, but less than 5% of notified suspected cases are sampled each year for culture. Although the biological data used in this study are aggregated annually and do not provide a clear indication of the spatiotemporal evolution of biological confirmation in the endemic and non-endemic provinces, nor do they distinguish the type of serotypes circulating in the country, the rate of collection of stool samples for biological analysis was very low (3.9%) from 2015 to 2021 with a *Vibrio cholerae* positivity rate of 25.9%. Biological surveillance should be strengthened by decentralizing biological analysis to the provinces.

A phylogenetic study has analyzed isolates collected in the DRC in 2011 and 2012 [57], when a large-scale epidemic started in the eastern provinces and rapidly spread across the country, affecting provinces in the west that had not experienced an epidemic for close to 10 years [58]. This study revealed that isolates grouped together as one discrete MLVA (Multi-Locus Variable Number Tandem Repeat Analysis) complex and that the epidemic was caused by the extensive expansion and diversification from a single MLVA haplotype [57]. Furthermore, isolates in the DRC were distinct from those collected in West Africa (Togo and Guinea) during the same timeframe [57]. Further phylogenetic studies of *Vibrio cholerae* strains circulating in the DRC should be conducted to clearly monitor the profiles and origins of *Vibrio cholerae* strains in each health zone (persisting bacterial populations vs. multiple re-introductions), establish the links and transmission pathways between separate outbreaks, and better understand the current disease dynamics. Such insight would further bolster cholera elimination strategies in the country.

Some study limitations should be noted. These analyses were conducted on data of suspected cholera cases collected by the disease surveillance and response system, which probably does not accurately reflect the real burden of cholera in the DRC because these data only take into account patients who consulted health facilities [59]. Nevertheless, a recent assessment of the surveillance data of diseases with epidemic potential tracked by the DRC has demonstrated that data on suspected cholera cases can be used for epidemiological or public health research purposes [23]. The collection of fecal samples for culture and case confirmation in some remote areas has been limited; however, since 2022, efforts have been made to improve the use of rapid diagnostic tests and PCR to strengthen surveillance in the country. The monitoring of funds mobilized for each plan has been limited and no economic evaluation has been conducted to quantify and monitor interventions implemented, hence the need to improve the coordination, management and financial assessment of cholera control in the DRC. Regarding the seasonal analysis, although the seasons vary between provinces that are located north and south of the equator, these variations do not seem to markedly influence our results as most cholera-endemic provinces are located south of the equator.

## Conclusion

The DRC implemented the first national cholera elimination plan in 2008. To date, three cholera elimination plans have been developed in the country (during 2008–2012, 2013–2017 and 2018–2021). Despite the implementation of the MCEPs, the spatiotemporal disease dynamics and seasonal cholera patterns have hardly improved, case numbers continue to peak at the end of the dry season and during the middle of the rainy season. Furthermore, the number of health zones categorized as cholera sanctuary zones has largely increased over time. This lack of progress in cholera elimination may be due to limitations in the method used to identify and prioritize sanctuary zones, which focused essentially on epidemiological parameters and some environmental indicators. To better prioritize high-risk health zones and orient epidemic prevention and preparedness activities, cholera sanctuary zones should be classified based on epidemiological, WASH, socio-economic, environmental and health indicators. Improvements should also be made regarding the strategy and implementation of the MCEP as well as the reporting and monitoring of funded activities. It is also essential to strengthen surveillance (including laboratory analysis of specimens) for a more rapid response, to lower the case fatality rate and to better understand the epidemiology of the disease. A similar study should be carried out at the health zone level. Additional studies on the cholera outbreak response in the DRC should also aim to identify bottlenecks and gaps at the local, national and international levels.

### Supplementary Information

**Additional file 1.** Context map of the Democratic Republic of the Congo. Provinces are labeled in bold, and lakes are indicated in italics.

**Additional file 2.** Annual proportion of cases and deaths in the DRC per province, before and during each MCEP period.

**Additional file 3.** Cholera attack rate in the DRC at the province level before and during each MCEP period (per 100,000 inhabitants).

**Additional file 4.** Lulls in cholera cases of more than four weeks in the endemic provinces.

## Data Availability

The data and R codes are available from the authors upon reasonable request and with the permission of the Head of the Department of Infectious Diseases Ecology Didier Bompangue.

## Abbreviations

DRC: Democratic Republic of the Congo
GTFCC: Global Task Force on Cholera Control
IDSR: Integrated Disease Surveillance and Response
MCEP: Multisectoral Cholera Elimination Plan
MLVA: Multi-Locus Variable Number Tandem Repeat Analysis
NIBR: National Institute of Biomedical Research
WASH: Water, sanitation and hygiene
WHO: World Health Organization
