# Supplementary material for: Spatiotemporal dynamics of cholera in the Democratic Republic of the Congo before and during the implementation of the Multisectoral Cholera Elimination Plan: a cross-sectional study from 2000 to 2021
Source: BMC Public Health. 2023 Aug 22;23:1592. doi: 10.1186/s12889-023-16449-2 (PMC10463990; doi:10.1186/s12889-023-16449-2)
Supplement: Supplementary file 1 — Additional file 1. Context map of the Democratic Republic of the Congo. Provinces are labeled in bold, and lakes are indicated in italics. [file 12889_2023_16449_MOESM1_ESM.docx]

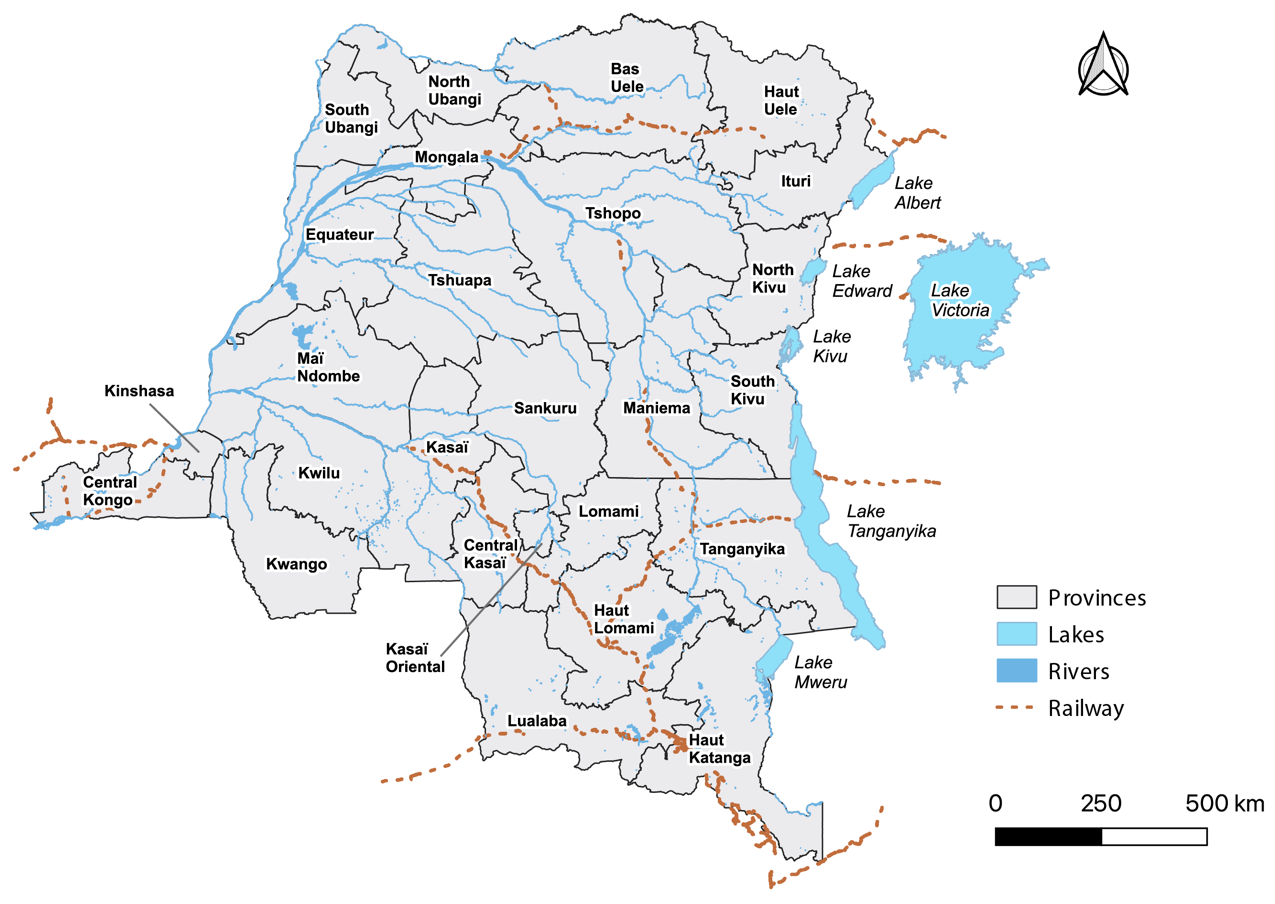


**Additional file 1. Context map of the Democratic Republic of the Congo.** Provinces are labeled in bold, and lakes are indicated in italics.
