# Supplementary material for: Spatiotemporal dynamics of cholera in the Democratic Republic of the Congo before and during the implementation of the Multisectoral Cholera Elimination Plan: a cross-sectional study from 2000 to 2021
Source: BMC Public Health. 2023 Aug 22;23:1592. doi: 10.1186/s12889-023-16449-2 (PMC10463990; doi:10.1186/s12889-023-16449-2)
Supplement: Supplementary file 2 — Additional file 2. Annual proportion of cases and deaths in the DRC per province, before and during each MCEP period. [file 12889_2023_16449_MOESM2_ESM.docx]

**Additional file 2. Annual proportion of cases and deaths in the DRC per province, before and during each MCEP period.**

| **Province** | **Pre-MCEP (2003-2007)** | | **MCEP-1 (2008-2012)** | | **MCEP-2 (2013-2017)** | | **MCEP-3 (2018-2021)** | |
| --- | --- | --- | --- | --- | --- | --- | --- | --- |
|  | **Total cases (%)** | **Total deaths (%)** | **Total cases (%)** | **Total deaths (%)** | **Total cases (%)** | **Total deaths (%)** | **Total cases (%)** | **Total deaths**  **(%)** |
| **Endemic provinces** | | | | | | | | |
| Haut Katanga | 6046 (6.1) | 213 (9.2) | 6999 (5.7) | 151 (7.6) | 14605 (10.3) | 332 (11.8) | 9578 (10.3) | 307 (15.0) |
| Haut Lomami | 16780 (16.9) | 572 (24.8) | 7258 (4.7) | 242 (12.2) | 13835 (9.7) | 324 (11.5) | 7199 (7.7) | 162 (7.9) |
| Ituri | 4220 (4.2) | 174 (7.5) | 5812 (4.7) | 181 (9.1) | 4537 (3.2) | 150 (5.3) | 866 (0.9) | 33 (1.6) |
| North Kivu | 19471 (19.6) | 447 (19.4) | 34617 (28.2) | 0 (0.0) | 27744 (19.6) | 209 (7.4) | 15751 (16.9) | 109 (5.3) |
| South Kivu | 28688 (28.8) | 252 (10.9) | 37214 (30.3) | 197 (9.9) | 33638 (23.7) | 131 (4.7) | 22992 (24.7) | 124 (6.1) |
| Tanganyika | 19055 (19.1) | 425 (18.4) | 13152 (10.7) | 83 (4.1) | 15517 (10.9) | 264 (9.4) | 14157 (15.2) | 184 (9.0) |
| **Total endemic provinces** | **94260 (94.7)** | **2083 (90.2)** | **105052 (85.5)** | **854 (43.0)** | **109876 (77.6)** | **1410 (50.0)** | **70543 (75.8)** | 919 (44.8) |
| **Non-endemic provinces** | | | | | | | | |
| Bas-Uele | 1 (0.0) | 0 (0.0) | 716 (0.6) | 29 (1.4) | 363 (0.3) | 11 (0.4) | 5 (0.0) | 1 (0.0) |
| Equateur | 9 (0.0) | 0 (0.0) | 2778 (2.3) | 86 (4.3) | 3734 (2.6) | 126 (4.5) | 906 (1.0) | 57 (2.8) |
| Haut-Uele | 0 (0.0) | 0 (0.0) | 437 (0.4) | 14 (0.7) | 1 (0.0) | 0 (0.0) | 1 (0.0) | 0 (0.0) |
| Kasai oriental | 2747 (2.8) | 120 (5.2) | 0 (0.0) | 0 (0.0) | 12 (0.0) | 1 (0.0) | 7839 (8.4) | 332 (16.2) |
| Kasai | 0 (0.0) | 0 (0.0) | 0 (0.0) | 0 (0.0) | 1384 (1.0) | 99 (3.5) | 1955 (2.1) | 131 (6.4) |
| Kinshasa | 7 (0.0) | 0 (0.0) | 1936 (1.6) | 37 (1.8) | 909 (0.6) | 66 (2.3) | 1355 (1.5) | 29 (1.4) |
| Kongo central | 280 (0.3) | 5 (0.2) | 1284 (1.0) | 15 (0.7) | 4438 (3.1) | 126 (4.5) | 1921 (2.1) | 93 (4.5) |
| Kasai central | 0 (0.0) | 0 (0.0) | 0 (0.0) | 0 (0.0) | 1 (0.0) | 0 (0.0) | 29 (0.0) | 0 (0.0) |
| Kwango | 28 (0.0) | 6 (0.3) | 0 (0.0) | 19 (0.9) | 1 (0.0) | 0 (0.0) | 0 (0.0) | 0 (0.0) |
| Kwilu | 12 (0.0) | 0 (0.0) | 301 (0.2) | 0 (0.0) | 672 (0.5) | 46 (1.6) | 429 (0.5) | 39 (1.9) |
| Lomami | 35 (0.0) | 7 (0.3) | 0 (0.0) | 87 (4.3) | 2401 (1.7) | 96 (3.4) | 1811 (1.9) | 123 (6.0) |
| Lualaba | 388 (0.4) | 37 (1.6) | 2426 (2.0) | 198 (9.9) | 590 (0.4) | 24 (0.9) | 1463 (1.6) | 58 (2.8) |
| Mai-Ndombe | 0 (0.0) | 0 (0.0) | 3722 (3.0) | 14 (0.7) | 1612 (1.1) | 72 (2.6) | 1369 (1.5) | 93 (4.5) |
| Maniema | 1615 (1.6) | 39 (1.7) | 560 (0.5) | 26 (1.3) | 6828 (4.8) | 165 (5.9) | 330 (0.4) | 15 (0.7) |
| Mongala | 0 (0.0) | 0 (0.0) | 544 (0.4) | 10 (0.5) | 3263 (2.3) | 205 (7.3) | 30 (0.0) | 2 (0.1) |
| Nord Ubangi | 0 (0.0) | 0 (0.0) | 0 (0.0) | 324 (16.3) | 443 (0.3) | 49 (1.7) | 1 (0.0) | 1 (0.0) |
| Sankuru | 130 (0.1) | 9 (0.4) | 0 (0.0) | 61 (3.0) | 507 (0.4) | 46 (1.6) | 2301 (2.5) | 144 (7.0) |
| Sud Ubangi | 1 (0.0) | 0 (0.0) | 837 (0.7) | 187 (9.4) | 12 (0.0) | 0 (0.0) | 10 (0.0) | 0 (0.0) |
| Tshopo | 19 (0.0) | 1 (0.0) | 1701 (1.4) | 21 (1.0) | 4495 (3.2) | 274 (9.7) | 662 (0.7) | 11 (0.5) |
| Tshuapa | 0 (0.0) | 0 (0.0) | 482 (0.4) | 0 (0.0) | 0 (0.0) | 0 (0.0) | 0 (0.0) | 0 (0.0) |
| **Total non-endemic provinces** | **5272 (5.2)** | **224 (9.7)** | **17724 (14.4)** | **1128 (56.9)** | **31666 (22.3)** | **1406 (49.9)** | **22417 (24.1)** | **1129 (55.1)** |
