# Supplementary material for: Spatiotemporal dynamics of cholera in the Democratic Republic of the Congo before and during the implementation of the Multisectoral Cholera Elimination Plan: a cross-sectional study from 2000 to 2021
Source: BMC Public Health. 2023 Aug 22;23:1592. doi: 10.1186/s12889-023-16449-2 (PMC10463990; doi:10.1186/s12889-023-16449-2)
Supplement: Supplementary file 4 — Additional file 4. Lulls in cholera cases of more than four weeks in the endemic provinces. [file 12889_2023_16449_MOESM4_ESM.docx]

**Additional file 4. Lulls in cholera cases of more than four weeks in the endemic provinces.**

|  | **Number of interruptions of more than four weeks** | | | |
| --- | --- | --- | --- | --- |
| **Endemic provinces** | **Pre-MCEP** | **MCEP-1** | **MCEP-2** | **MCEP-3** |
| South Kivu | 0 | 0 | 1(5,5) | 0 |
| North Kivu | 0 | 0 | 1(5,5) | 0 |
| Tanganyika | 0 | 0 | 0 | 0 |
| Haut-Lomami | 2 (7,9) | 2(7,9) | 2(5,9) | 1(7,7) |
| Haut-Katanga | 7(5,11) | 7(5,11) | 4(6,10) | 5(5,14) |
| Ituri | 8(5,34) | 7(5,35) | 6(5,22) | 6(15,32) |

The minimum and maximum number of weeks with 0 cases are indicated in parentheses.
